# Oral Vaccination with Attenuated *Salmonella* Expressing Viral M25 Protein Effectively Protects Mice Against Murine Cytomegalovirus Infection

**DOI:** 10.3390/pathogens14040314

**Published:** 2025-03-25

**Authors:** Yujun Liu, Hao Gong, Jiaming Zhu, Fenyong Liu

**Affiliations:** 1 School of Public Health, University of California, Berkeley, CA 94720, USA; 2 Program in Comparative Biochemistry, University of California, Berkeley, CA 94720, USA

**Keywords:** congenital infection, cytomegalovirus, gene delivery, herpesvirus, oral vaccine, *Salmonella*, vaccines

## Abstract

Attenuated *Salmonella* strains are promising oral vectors for vaccination against human infectious diseases. Human cytomegalovirus (CMV) is among the most common causes of disability in children, including intellectual disability and sensorineural hearing loss. Developing an anti-CMV vaccine is a major public health priority. We report in this study the construction of a new attenuated *Salmonella* strain to express murine cytomegalovirus (MCMV) M25 protein and its use for vaccination in mice against MCMV infection. In mice orally vaccinated with the constructed *Salmonella* vector carrying the M25 expression cassette, we revealed a substantial induction of anti-MCMV serum IgG and mucosal IgA humoral responses and a considerable elicitation of anti-MCMV T cell responses. When the vaccinated mice were challenged intraperitoneally and intranasally with MCMV, we observed a significant inhibition of virus infection and growth in various organs including spleens, livers, lungs, and salivary glands, compared to the non-vaccinated animals or those receiving a control vaccine without M25 protein expression. Moreover, we showed effective protection of these vaccinated mice from MCMV challenge. Our study provides the first direct evidence that an attenuated *Salmonella*-based vector with the MCMV M25 expression cassette can induce strong humoral and T cell responses and provide effective protection against MCMV infection. These results illustrate the feasibility of engineering *Salmonella*-based vectors expressing the M25 antigen for anti-CMV oral vaccine development.

## 1. Introduction

Developing a vaccine against human cytomegalovirus (CMV) represents a major public health priority [1,2]. This is because this virus, also called HCMV, is among the most common infectious causes of disability in children, including intellectual disability and sensorineural hearing loss [3]. Moreover, HCMV infection causes significant morbidity and mortality in immunodeficient individuals such as organ transplant and hematopoietic stem cell transplant patients, as well as AIDS patients [4]. It has been shown that HCMV is ubiquitous worldwide, affecting 1 in 71 in low- and middle-income countries [5,6]. While there is currently no FDA-approved vaccine against HCMV infection, tremendous efforts on anti-HCMV vaccines have been and are currently being made and have shown much promise [1,2].

Because HCMV is species-specific [4], animal models of infection by nonhuman CMVs such as murine CMV (MCMV) infection in mice have been used to provide insight into our understanding of HCMV infection in humans and to help develop vaccines against CMV infection [7,8]. A wide array of anti-CMV vaccines, such as virus-like particle (VLP) or subviral dense body vaccines, inactivated whole-viral-antigen vaccines, DNA vaccines, recombinant protein vaccines, and vaccines based on live attenuated virus vectors, have been tested in animals and humans [1,2,9]. In particular, some of these vaccines, when tested in mice challenged with MCMV, induced excellent immune responses and provide immune protection from MCMV challenge [10,11,12,13,14]. These results illustrate the use of MCMV infection of mice as a model for developing vaccines against CMV infection and associated diseases.

Orally administered vaccines have significant advantage for mass vaccination compared to vaccination through injection administration. Attenuated *Salmonella* strains have been constructed and used as oral gene delivery vectors for gene therapy and vaccinations [15,16,17]. Once transformed with plasmid constructs of transgenes, the attenuated bacterial mutant strains can enter the infected cells and deliver the plasmid DNA, resulting in transgene expression [18,19]. It has been demonstrated that bacterial virulence factors are important for *Salmonella* intracellular survival, a critical step for efficient *Salmonella*-mediated gene transfer [20,21], and that inactivation of several *Salmonella* virulence factors increases the gene delivery ability of the constructed vectors [19,22].

In this report, we constructed a new attenuated *Salmonella* strain, S535, with a deletion at the *msbB* gene. The msbB protein is an enzyme involved in the production of lipopolysaccharide (LPS), a major bacterial virulence and proinflammatory factor [21]. Inactivating msbB would attenuate the bacterial virulence/toxicity and improve intracellular bacterial lysis, resulting in better transgene delivery and expression. In the current study, a *Salmonella*-based vaccine, S-M25, was derived from S535 with a construct expressing MCMV M25 protein. M25 and its HCMV homolog, UL25, are found in viral infectious particles. While our laboratory and other laboratories have shown that both genes are dispensable for viral replication in vitro, M25 and UL25 are known to play multiple functional roles in viral infections, such as affecting the cytopathic effects, sequestering the tumor suppressor protein p53 in nuclear accumulations, interacting with cellular targets, and modulating innate immune responses [23,24,25,26,27,28]. Both UL25 and M25 are virion proteins and major targets for anti-CMV antibodies during natural viral infection [29,30,31], therefore representing ideal antigens for anti-CMV vaccine development. However, whether M25 or UL25 can serve as an antigen for vaccine development has not been studied.

Our study reported here reveals the first direct evidence that an attenuated *Salmonella*-based vector with the MCMV M25 expression cassette can induce strong humoral and T cell responses against MCMV infection in vaccinated mice. Moreover, immunization with S-M25 provides immune protection and inhibits viral replication and infection in vaccinated mice intraperitoneally and intranasally challenged with MCMV. Our study demonstrates the feasibility of using *Salmonella*-based vectors expressing the M25 antigen for anti-CMV oral vaccine development.

## 2. Materials and Methods

### 2.1. Institutional Review Board Statement

Our study was performed following the recommendations of the Guide for the Care and Use of Laboratory Animals of the National Research Council. The Animal Care and Use Committee of the University of California at Berkeley approved our animal experiment protocol (Protocol #R240) on 7 December 2022.

### 2.2. Viruses, Cells, and Plasmid Constructs

We followed the previously described protocols to maintain cultures of mouse NIH 3T3 cells and J774 macrophages (American Type Culture Collection (ATCC), Manassas, VA, USA). We used the BAC-mid approach [23,32,33,34] to generate mutant m-M25, which contained a deletion of the M25 coding sequence (coordinates 26015–28810) (accession number U68299) [35]. We confirmed the M25 deletion with restriction digestion mapping and sequencing analyses of the m-M25 genomic DNA. We propagated the MCMV Smith strain (ATCC, catalog number VR-194) and mutant m-M25 in NIH3T3 cells, following previously described procedures [36,37]. We constructed the M25 expression plasmid peM25 by cloning the M25 coding sequence into vector peVAX, a derivative of pVAX (Invitrogen, Carlsbad, CA, USA) with additional restriction enzyme cloning sites, a CMV major immediate-early gene promoter, and a kanamycin resistance gene for selection in *E. coli*. The M25 coding sequence was obtained by PCR using the Smith strain DNA as the template in the presence of 5′ primer 5M25 (5′-CCGGAATTCGGTACCAGCCAGTTCGTACAGCACGT-3′) and 3′ primer 3M25 (5′-CGCGGATCCGGTACCTTACAGAAAGGTACGCTTGGA-3′).

### 2.3. Salmonella-Based Vaccines and M25 Expression

We deleted the coding sequence of the *msbB* gene from *Salmonella typhimurium* aroA strain SL7207 (a gift from Bruce A. D. Stocker, Stanford University, Stanford, CA, USA) [38] to create attenuated strain S535, following the λ Red recombinase-based mutagenesis procedure [39]. In the mutagenesis procedure, the PCR products for the msbB deletion were produced with the template pKan-clone7 in the presence of 5′ primer 5msbB (5′-GACGCAGACGTCGCTACACTATTCACAATTCCTTTTCGCGTCAGCAGACCCTGGAAAGCCATATGAATATCC TCCTTAGTTC-3′) and 3′ primer 3msbB (5′-CCTGTTCATCAGGTAGTACAGGGTTTGTCAGCATAAAGCCTCTCTTACGAGAGGCTTTATTGTGTAGGCTGGAGCTGCTT-3′) and transformed into SL7207 to create S535 following the described methods [40,41].

Constructs peM25 and peVAX were transformed to S535 to give rise to vaccines S-M25 and S-vec, respectively. In vitro growth (in LB broth) of bacterial strains was performed as described previously [40,41]. To examine M25 expression from vaccine S-M25, we first treated mouse J774 macrophages with IFN-γ (150 U/mL) (R&D Systems Inc., Minneapolis, MN, USA) and then infected them with S-M25 and S-vec (MOI = 0.5). Western blot analyses of samples isolated from cells at 72 h postinfection were conducted following the previously described procedures [40], using a polyclonal rabbit anti-M25 antibody generated with M25 peptides (Promab, Inc., Richmond, CA, USA).

### 2.4. Oral Immunization of Mice

After being anesthetized, four-week-old BALB/c mice (Jackson Laboratory, Bar Harbor, ME, USA) were intragastrically inoculated with phosphate-buffered saline (PBS) containing no *Salmonella* or 1 × 10^9^ cfu S-M25 or S-vec [40,41]. Animals were immunized on days 0, 14, and 28. For each experiment, two trials were conducted with 5 mice per group.

### 2.5. MCMV-Infected Cell Lysate ELISA

At indicated time points, we collected mucosal nasal wash and serum samples from mice as described previously [42,43]. Specifically, we collected blood samples and transferred them to microtainer tubes (Becton Dickinson, San Jose, CA, USA) following the manufacturer’s recommendations. We collected and prepared the nasal wash samples following the procedures described previously [43]. We used the cell lysates infected with wildtype MCMV Smith strain or mutant m-M25 to assay the anti-MCMV IgG and IgA antibody reactivity of the collected samples. The cell lysates were harvested and prepared from NIH3T3 cells infected with the Smith strain or mutant m-M25 (MOI = 1) at 4 days postinfection and quantified by measuring absorbance at 280 nm in a spectrophotometer [36,37].

ELISA assays were employed to measure the reactivity of the serum anti-MCMV IgG and IgA antibody against cell lysates infected with wildtype MCMV Smith strain or mutant m-M25. In the ELISA assay, each well of 96-well Medisorp ELISA plates (Thermo Fisher, Waltham, MA, USA) was coated with 100 µL of 50 µg/mL cell lysate diluted in coating buffer (50 mM carbonate–bicarbonate buffer, 1.59 g Na_2_CO_3_ and 2.93 g NaHCO_3_ in 1 L H_2_O, pH 9.4), sensitized overnight at 4 °C, and then washed by adding 200 µL of ELISA wash buffer (0.05 M Tris, 0.138 M NaCl, 0.0027 M KCl, 0.05% Tween-20). After washing, the wells of the ELISA plate were blocked with 200 µL per well of 5% non-fat powdered milk in PBS, washed, and added with 100 µL of serum diluted in ELISA dilution buffer (5% Bovine Serum Albumin, BSA; VWR, Randor, PA, USA). The plate was then incubated for one hour on an orbital shaker, once again washed, and reacted with 100 µL of goat anti-mouse IgG (H+L) (Cell Signaling Technologies, Danvers, MA, USA) per well (1:2000 dilution) for one hour on an orbital shaker. The plate was washed, mixed with a chemiluminescent substrate (BioLegend, California, San Diego, CA, USA), and analyzed in a Spectramax plate reader (San Jose, CA, USA), following the manufacturer’s recommendations. We conducted each assay in duplicate and repeated each experiment three times.

### 2.6. T Cell ELISPOT Assay

Mouse IFN-γ ELISPOT kits (U-Cytech biosciences, Utrecht, The Netherlands) were applied to assay the number of T cells that expressed IFN-γ [44]. Following the manufacturer’s recommendations, we incubated splenocytes (1 × 10^6^ cells) with Smith- or m-M25-infected cell lysates (150 µg/well) in the ELISPOT plates, which were then reacted with an antibody against IFN-γ and finally analyzed using a camera [44]. We conducted each assay in duplicate and repeated each experiment three times.

### 2.7. MCMV Challenge Experiments of Vaccinated Animals

Fourteen days after the final vaccination, we intraperitoneally or intranasally infected groups of mice (5–10 animals per group) with salivary gland-passaged MCMV Smith strain (1 × 10^6^ PFU per mice) (for lethal dosage challenge with an LD_50_ of 1 × 10^5^ PFU) or cultured cell-passaged MCMV Smith strain (5 × 10^4^ PFU per mice) [36,37]. Mice receiving MCMV lethal dosage were monitored daily for two weeks, and their survival was recorded, following established guidelines in the Guide for the Care and Use of Laboratory Animals to maximize the animal’s comfort [36,37].

Mice receiving MCMV sublethal dosages were sacrificed at 5 days post challenge, and spleens, livers, lungs, and salivary glands were harvested [36,37]. Virus samples were prepared from these organs and the MCMV titers in these samples were determined in NIH 3T3 cells using the plaque assays as described previously [36,37]. We conducted each assay in duplicate and repeated each experiment three times.

### 2.8. Statistical Analysis

We conducted all assays in duplicate and repeated each experiment three times. We performed statistical analyses using GraphPad Prism software (version 10) and considered a *p*-value of less than 0.05 as significant.

## 3. Results

### 3.1. Studies of Attenuated Salmonella Expressing MCMV M25 Protein

Attenuated *Salmonella* vectors were developed for delivery of functional RNAs such as ribozymes for antiviral application in our laboratory [19]. We here report the construction of a novel attenuated *Salmonella* strain, S535, which was derived from the auxotrophic *Salmonella typhimurium* aroA strain SL7207 [38] with a deletion of the *msbB* gene. The msbB protein is an enzyme involved in the production of lipopolysaccharide (LPS), a major bacterial virulence and proinflammatory factor [21]. We previously showed that SL7207 delivered expression cassettes of functional RNAs such as ribozymes for transgene expression in mouse cells [19]. Thus, S535 is expected to be more attenuated and exhibit less virulence due to the deletion of the *msbB* gene compared to SL7207.

Two vaccines were constructed for this study. The first vaccine, S-M25, was derived from S535 with the transformation of the M25-expressing construct peM25. In peM25, the M25 expression is driven by a eukaryotic expression promoter. The second vaccine, S-vec, which served as the negative control, was derived from S535 with the transformation of the empty expression vector construct peVAX without the M25 sequence.

**Figure 1:**
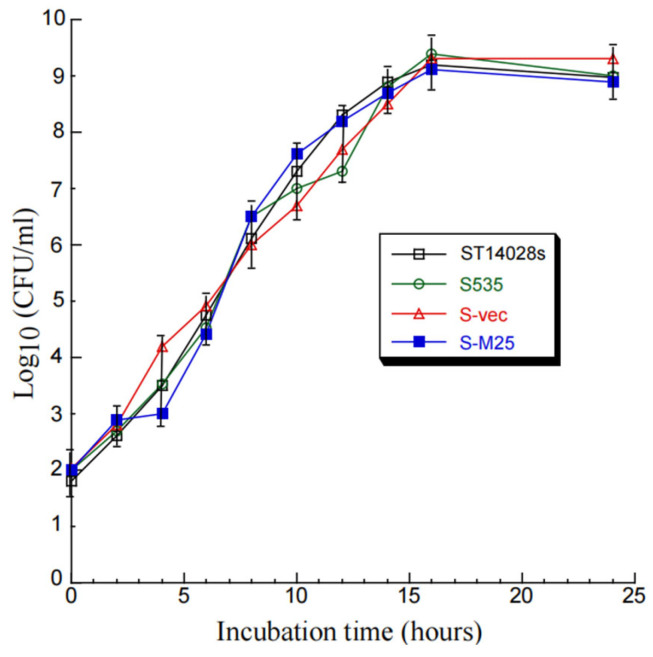
In vitro growth (in LB broth) of *Salmonella* clinical strain ST14028s, mutant S535, and S535 with the empty vector peVAX (S-vec), and the M25 expression construct peM25 (S-M25).

**Figure 2:**
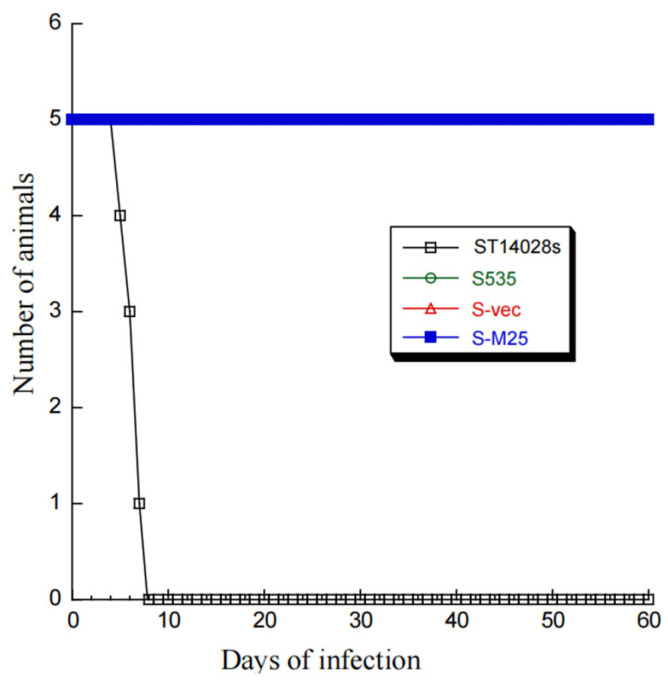
A 60-day time course of BALB/c mice’s survival after intragastric infection by *Salmonella* strains ST14028s (2 × 10^3^ CFU/mice), S535 (1 × 10^9^ CFU/mice), S-vec (1 × 10^9^ CFU/mice), and S-M25 (1 × 10^9^ CFU/mice).

Compared to parental strain S535 and a clinical strain ST14028s, the presence of the vector and M25 sequences appeared to have no effect on the growth and viability of S-M25 and S-vec. This is because S-M25 and S-vec grew well as S535 and ST14028s in LB broth in vitro (Figure 1). The ability of these *Salmonella* strains to kill mice was also assessed (Figure 2). Among mice infected with clinical strain ST14028s (2 × 10^3^ cfu/mouse), no survival was found beyond 7 days postinfection. In contrast, all mice infected with S535, S-vec, and S-M25 remained alive even though the animals were infected with a higher dose of bacteria (1 × 10^9^ cfu/mouse) and examined up to 60 days postinfection (Figure 2). Thus, S-M25 and S-vec appeared to be substantially attenuated and exhibit little virulence to kill mice in vivo.

The constructed attenuated *Salmonella*-based vaccine S-M25 appeared to express M25 protein in infected mouse J774 macrophages. Western blot analyses detected the expression of the M25 protein of ~130 kDa in J774 cells infected with S-M25 but not control vaccine S-vec, which contained only the empty expression vector without any MCMV sequence (Supplementary Materials, Figure S1). We did not detect M25 expression in S-M25 grown in LB broth in the absence of J774 cells, because the M25 expression was under the control of a eukaryotic promoter and only occurred inside mammalian cells.

### 3.2. Studies of Salmonella-Based Vaccine-Induced Humoral and T Cell Responses

We intragastrically treated groups of mice (5 mice per group) with phosphate-buffered saline (PBS) or vaccinated them with S-vec and S-M25 at days 0, 14, and 28. All mice remained alive up to 42 days post-immunization when the experiments were terminated two weeks after the final immunization. These results further confirm our observations of the lack of ability of the constructed *Salmonella* strain S535 and its derived vaccines S-vec and S-M25 to kill mice (Figure 2), probably due to their low virulence/pathogenicity in vivo.

**Figure 3:**
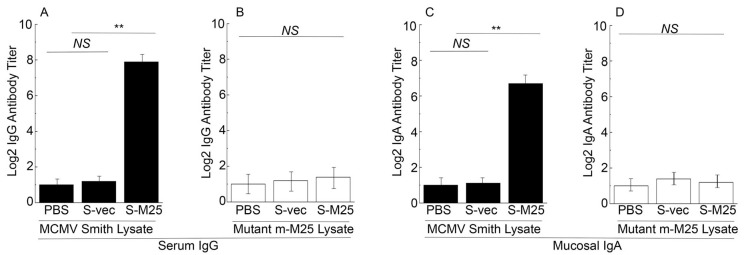
The titers of serum IgG and mucosal IgA from the intragastrically vaccinated mice. Animals were treated with PBS only, control vaccine S-vec, and M25-expressing vaccine S-M25 at days 0, 14, and 28, and pool serum or mucosal wash samples were collected from mice as described previously [42,43]. ELISA assays were performed to analyze serum IgG titers against lysates of cells infected with MCMV Smith (**A**) and mutant m-M25 (**B**), and mucosal IgA titers against lysates of cells infected with MCMV Smith (**C**) and mutant m-M25 (**D**). ** *p* < 0.05. NS, not significant. We performed the assays in duplicate and repeated them three times. The results are the average of the three experiments.

Serum antibodies collected from mice were assayed using ELISA to detect the presence of anti-M25 antibodies (Figure 3). Two antigen samples were used in our ELISA assays. The first sample included the lysates from cells infected with the wildtype MCMV Smith strain. The second sample was the lysates from cells infected with mutant m-M25, which was generated from the Smith strain by deleting the M25 open reading frame. Previous studies from our and other laboratories reported that M25 was dispensable for MCMV replication in vitro and M25-minus MCMV mutants grew well as the Smith strain in NIH3T3 cells [24,25]. The sample with m-M25 was used as a control to determine if the humoral responses from the immunized animals were specific against M25 since this mutant did not express M25 due to the deletion of the M25 sequence.

When the lysates from the Smith-infected cells were used as the antigen source, the ELISA showed about 200-fold higher antibody titers in sera from S-M25-vaccinated mice than in sera from S-vec-vaccinated mice (Figure 3A). In contrast, when the lysates from the m-M25-infected cells were used as the antigen source, the ELISA detected low antibody titers from the sera of the S-M25- and S-vec-vaccinated animals (Figure 3B). Thus, the constructed *Salmonella*-based vaccine S-M25 appeared to elicit strong and M25-specific IgG humoral responses.

*Salmonella* and its derived vaccine vectors are known to induce mucosal immune responses due to its inoculation and infection of the gastrointestinal tract [45]. We conducted ELISA assays to study if the constructed vaccines also elicit mucosal antibody responses. Specifically, we assayed the anti-MCMV IgA titers from the nasal wash obtained from the vaccinated mice at 42 days post-immunization. Both the lysate samples from cells infected with the Smith strain and the mutant m-M25 were used in our ELISA experiments. Low IgA titers were detected from the vaccinated mice in ELISA experiments using the lysates with m-M25 (Figure 3D). In contrast, the IgA titers from S-M25 vaccinated mice were approximately 50-fold higher than those in S-vec vaccinated mice in ELISA experiments using the lysates with the Smith strain (Figure 3C). Thus, the constructed *Salmonella*-based vaccine S-M25 also appeared to elicit M25-specific IgA humoral responses.

**Figure 4:**
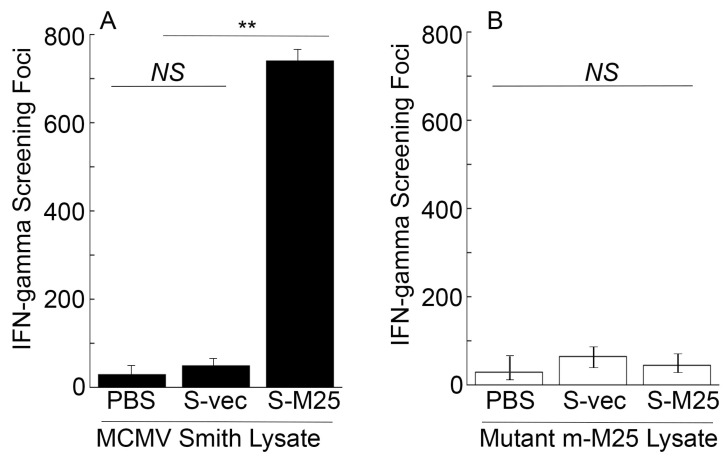
Cellular mediated immune responses from the intragastrically vaccinated mice. Animals were treated with PBS only, control vaccine S-vec, and M25-expressing vaccine S-M25 at days 0, 14, and 28. Splenocytes (*n* = 5) were collected at 42 days post-immunization and exposed to Smith-infected (**A**) and m-M25-infected cellular lysates (**B**) for 48 h. ELISPOT assays were performed to determine the number of IFN-γ-producing T cells, represented as spot-forming cells (SFCs) per million cells. ** *p* < 0.05. NS, not significant. We performed the assays in duplicate and repeated them three times. The results are the average of the three experiments.

To assess the T cell responses elicited by the vaccines, splenocytes were collected from mice 42 days post-immunization and allowed to react with the Smith-infected and m-M25-infeced cell lysates. In experiments with the Smith-infected cell lysates, we observed about 80 times higher anti-MCMV IFN-γ-producing T cell responses in mice vaccinated with S-M25 than those vaccinated with S-vec (Figure 4A). On the contrary, we observed little anti-MCMV T cell response in all vaccinated mice in experiments with the m-M25-infected cell lysates (Figure 4B). Thus, vaccine S-M25 appeared to elicit M25-specific T cell responses.

### 3.3. Immune Protection of S-M25-Vaccinated Mice from Intraperitoneal and Intranasal MCMV Challenge

A series of experiments were performed to investigate if vaccination with S-M25 provides immune protection against systemic and mucosal MCMV challenge, respectively. In these experiments, we administered mice with PBS, S-vec, and S-M25 at days 0, 14, and 28, and then challenged the animals intraperitoneally or intranasally with lethal doses of salivary gland-passaged highly pathogenic MCMV at day 42 post-administration.

**Figure 5:**
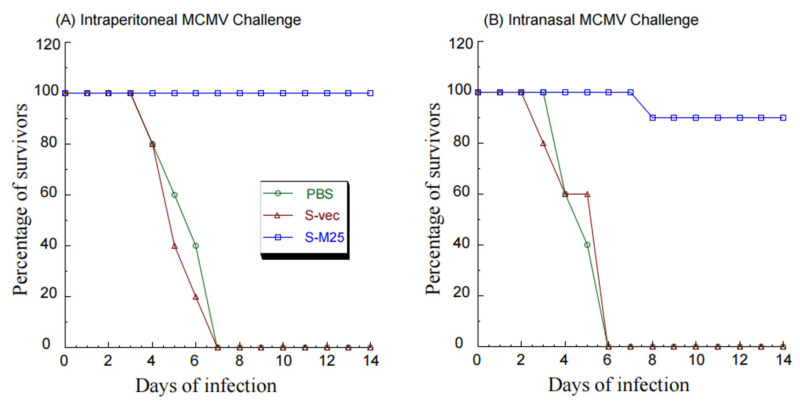
A 14-day time course of BALB/c mice’s survival after intragastrical treatment with PBS only, control vaccine S-vec, and M25-expressing vaccine S-M25 three times at days 0, 14, and 28. At 42 days post initial immunization, we intraperitoneally (**A**) or intranasally (**B**) infected the vaccinated animals with salivary gland-passaged MCMV Smith (1 × 10^6^ PFU per mice) and observed the animals daily for their survival.

In intraperitoneally challenged animals, S-M25 offered 100% protection after 14 days post challenge, while PBS and S-vec provided no protection. This was evident because the S-M25 vaccinated mice survived, and all animals administered with PBS or S-vec died within 7 days post challenge (Figure 5A). Similarly, in intranasally challenged animals, S-M25 offered 90% protection after 14 days post challenge, while PBS and S-vec provided no protection, as our results showed 90% survival of S-M25-vaccinated mice and 0% survival of PBS-treated or S-vec-vaccinated mice (Figure 5B).

**Figure 6:**
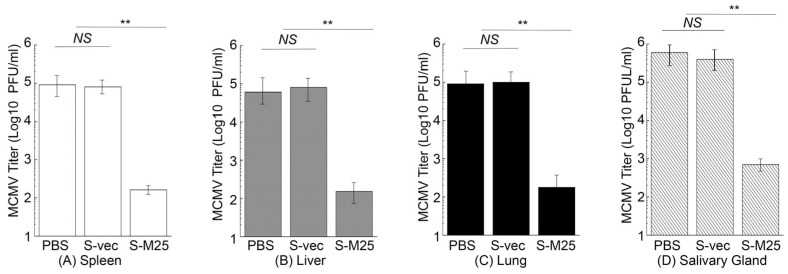
MCMV growth in the vaccinated mice after intraperitoneal viral challenge. Animals were intragastrically treated with PBS only, control vaccine S-vec, and M25-expressing vaccine S-M25 at days 0, 14, and 28, and then challenged with MCMV Smith (5 × 10^4^ PFU) at 42 days post-immunization. We assayed the viral titers in spleens (**A**), livers (**B**), lungs (**C**), and salivary glands (**D**) collected at 5 days post challenge. ** *p* < 0.05. NS, not significant. We performed the assays in duplicate and repeated them three times.

**Figure 7:**
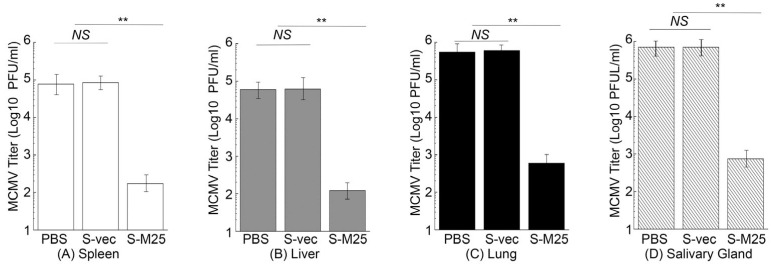
MCMV growth in the vaccinated mice after intranasal viral challenge. Animals were intragastrically treated with PBS only, control vaccine S-vec, and M25-expressing vaccine S-M25 at days 0, 14, and 28, and then challenged with MCMV Smith (5 × 10^4^ PFU) at 42 days post-immunization. We assayed the viral titers in spleens (**A**), livers (**B**), lungs (**C**), and salivary glands (**D**) collected at 5 days post challenge. ** *p* < 0.05. NS, not significant. We performed the assays in duplicate and repeated them three times.

In another series of experiments, we further examined virus growth in the MCMV-challenged mice immunized with these vaccines in order to examine the vaccine-induced immune protection. In these experiments, we administered mice with PBS, S-vec, and S-M25 at days 0, 14, and 28, and then challenged the animals intraperitoneally or intranasally with sublethal doses of MCMV at day 42 post-administration. We collected the spleens, livers, lungs, and salivary glands from mice at 5 days post challenge and determined the viral titers. In intraperitoneally challenging experiments, a reduction of 500-, 400-, 500-, and 850-fold was found in viral titers from the spleens, livers, lungs, and salivary glands of the S-M25-vaccinated mice, compared to those of the PBS-treated mice, respectively (Figure 6). It is noted that we observed substantial levels of viral titers in these organs from the S-vec mice, which were not significantly different from those in mice treated with PBS. Similar results were also observed in intranasally challenging experiments, as a reduction of 450-, 450-, 950-, and 950-fold was found in viral titers from the spleens, livers, lungs, and salivary glands of the S-M25-vaccinated mice, compared to those of the PBS-treated or S-vec-vaccinated mice, respectively (Figure 7). These results imply that the vaccination with *Salmonella*-based vaccine S-M25 provides immune protection and inhibits viral replication in mice intraperitoneally and intranasally challenged with MCMV.

## 4. Discussion

An anti-HCMV vaccine is urgently needed and is considered a major public health priority [1,2] because of the ubiquitous nature of the HCMV infection and the devastating complications associated with HCMV infection such as congenital CMV diseases. Although tremendous efforts on anti-HCMV vaccines have been made and have shown promising progress, no FDA-approved vaccines are currently available against HCMV infection [1,2].

Oral administration represents a significant advantage for mass vaccination compared to vaccination through injection administration. Our laboratory has previously explored the use of attenuated *Salmonella* strains as gene delivery vectors for the expression of functional RNAs such as ribozymes for antiviral applications [19]. In this report, we constructed a new attenuated *Salmonella* strain, S535, with a deletion at the *msbB* gene, which encodes an enzyme to produce lipopolysaccharide (LPS), a major bacterial virulence and proinflammatory factor [21]. Inactivating msbB would attenuate the bacterial virulence and reduce their toxicity and pathogenicity, increasing the bacterial capability as gene delivery vectors for transgene delivery and expression. We constructed a *Salmonella*-based vaccine, S-M25, which was derived from S535 with a construct expressing MCMV M25 protein. S535 and its derived vaccines, S-M25 and S-vec, displayed no defects in growth in LB broth in vitro (Figure 1). Furthermore, they exhibited little virulence in killing mice in vivo, compared to the clinical strain ST14028s (Figure 2). Mice vaccinated with S-M25 displayed an induction of anti-MCMV serum IgG and mucosa IgA levels and anti-MCMV T cell responses, compared to those animals treated with PBS or the control vaccine S-vec (Figure 3 and Figure 4). When challenged with MCMV, the S-M25-vaccinated animals exhibited a reduction in viral infection and replication in various organs and an increase in survival rate (Figure 5, Figure 6 and Figure 7).

Interestingly, we observed a greater reduction in lung viral titers following sublethal intranasal challenge compared to intraperitoneal challenge (Figure 6 and Figure 7), suggesting that the anti-MCMV mucosal immunity induced by oral immunization of the *Salmonella*-based vaccine is effective against mucosal MCMV challenge, leading to a greater reduction in viral infection at the mucosal inoculation site. However, less protection was found in mice intranasally challenged compared to those intraperitoneally challenged (Figure 5). This could mean that viral titers in the lungs of the intranasally challenged mice were still higher than those of the intraperitoneally challenged mice (comparing Figure 6C and Figure 7C). More importantly, our study reported here provides the first direct evidence that an attenuated *Salmonella*-based vector with the MCMV M25 expression cassette can induce strong immune responses and effective immune protection against MCMV infection in vaccinated mice. Therefore, attenuated *Salmonella* strain S535 can be used as a novel oral gene delivery vector for the construction of an anti-CMV vaccine.

Possible safety concerns need to be considered when live *Salmonella* is used for vaccination. While our vaccination experiments with a high dose (~1 × 10^9^ cfu/mice) of bacteria elicited strong immune responses and effective immune protection, it is more ideal to use lower doses of bacteria to prevent potential side effects and safety concerns. Future experiments using different and lower doses of bacteria will reveal the optimal doses that induce effective immune responses and protection against MCMV. We also need to understand the potential turnover and clearance of the bacteria post-immunization. Meanwhile, the exact mechanism of how attenuated *Salmonella* vectors deliver the transgenes for efficient expression in cells is not completely understood. An attenuated *Salmonella* strain has been approved as the vaccine for the prevention of typhoid fever [46,47]. Additional studies are needed to address these issues. For example, the generation of novel *Salmonella* mutants with new mutations affecting their virulence and pathogenicity, their immunogenicity and clearance by the immune system, and their ability for better gene delivery and transgene expression will facilitate the application of *Salmonella*-based vectors for vaccine development.

A wide array of anti-CMV vaccines, such as virus-like particle (VLP) or subviral dense body vaccines, inactivated whole-viral-antigen vaccines, DNA vaccines, recombinant protein vaccines, and vaccines based on live attenuated virus vectors, have been tested in animals and humans [1,2,9]. In particular, some of these vaccines, when tested in mice challenged with MCMV, induced excellent immune responses and provided immune protection against MCMV challenge [10,11,12,13,14]. However, whether M25 or UL25 can serve as an antigen for vaccine development has not been reported. Both UL25 and M25 are virion proteins and the major targets of anti-CMV antibodies during natural viral infection [29,30,31], therefore representing ideal antigens for anti-CMV vaccine development. While both genes are dispensable for viral replication in vitro, M25 and UL25 play multiple functional roles in viral infections, such as sequestering the tumor suppressor protein p53 in nuclear accumulations, affecting the cytopathic effects, interacting with cellular targets, and modulating innate immune responses [23,24,25,26,27,28]. Moreover, a subviral dense body vaccine, which was derived from the HCMV Towne strain with the deletion of the UL25 sequence, is currently being explored [9]. Our results showed that vaccine S-M25 elicited strong humoral and T cell responses against MCMV infection in vaccinated mice. Moreover, immunization with S-M25 provides immune protection and inhibits viral replication and infection in vaccinated mice intraperitoneally and intranasally challenged with MCMV.

We recently constructed a *Salmonella* vaccine vector with the *ssrA/B* mutation and showed that mice, when orally immunized with this vector expressing the MCMV M78 antigen, exhibited induced anti-MCMV humoral and T cell immune responses and were protected from MCMV challenge [48]. Our current study, which used a different *Salmonella* mutant (i.e., with the *msbB* mutation) expressing a different MCMV antigen (i.e., M25), showed similar levels of elicited immune responses and immune protection in immunized mice. Together, these studies illustrate the feasibility of using *Salmonella*-based vectors expressing viral antigens for anti-CMV oral vaccine development.

We note that immune responses to CMV infection in humans may be very different from those in mice, as described in this study [4,7,8]. Additional studies are needed to investigate how *Salmonella*-based vaccines expressing different viral antigens elicit immune responses and provide immune protection in humans. We also note that our study represents the first series of experiments to study the potential of S535-based vectors expressing viral antigens for anti-CMV vaccine development, and many additional experiments are needed. For example, the levels of key cytokines (e.g., IL-2 and IL-4) in the immunized mice should be assessed to understand the induced anti-MCMV T cell immunity. The M25 protein/peptide sequences and epitopes recognized by the serum IgG and mucosal IgA antibodies should be determined to understand how these antibodies react and bind to M25 protein. Moreover, whether these antibodies can affect viral infection and replication should be investigated. However, these antibodies are not believed to bind to intact infectious virions and neutralize their infectivity because M25 is only localized within the tegument inside the intact virions and is not expressed on the virion surface [29,30,31]. Another series of important and interesting experiments are to determine the levels of immune responses and immune protection in vaccinated mice when they are challenged by MCMV both intraperitoneally and intranasally. These studies will provide further insights into our understanding of the immune responses elicited by the *Salmonella*-based vaccine expressing M25 protein. We hope that these results will lead to the development of an effective vaccine for the prevention of HCMV infection.

## Data Availability

The dataset is available on request from the authors.

## Supplementary Materials

The following supporting information can be downloaded at https://www.mdpi.com/article/10.3390/pathogens14040314/s1: Figure S1: M25 expression detected in mouse J774 cells infected with vaccines S-vec and S-M25 by Western blot analyses.
