# Supplementary figures and images for: Oral Vaccination with Attenuated Salmonella Expressing Viral M25 Protein Effectively Protects Mice Against Murine Cytomegalovirus Infection
Source: Pathogens. 2025 Mar 25;14(4):314. doi: 10.3390/pathogens14040314 (PMC12030445; doi:10.3390/pathogens14040314)

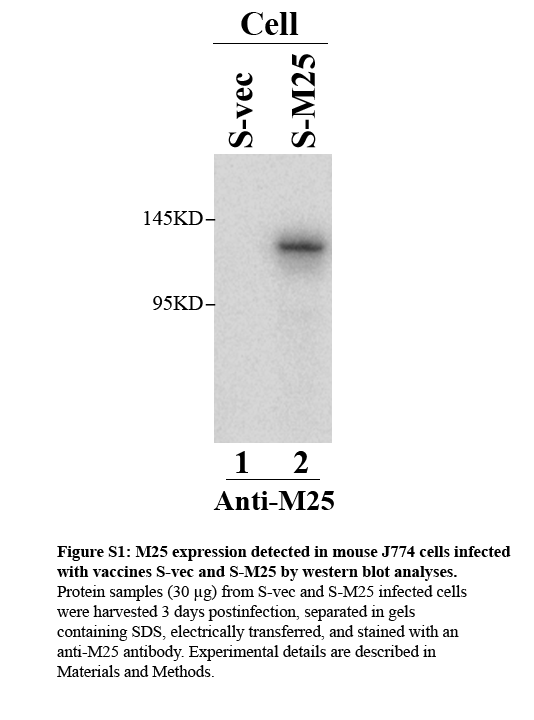

Supplement: Supplementary file 1 [file pathogens-14-00314-s001.zip › pathogens-3516769-Supplementary Material FigureS1.tif]
